# Association between advanced lung cancer inflammation index and all-cause and cause-specific mortality among asthma patients: a cohort study

**DOI:** 10.3389/fnut.2025.1519271

**Published:** 2025-02-06

**Authors:** Tu-Lei Tian, Xiang-Kun Qu, Hong-Bo Zhang, Cun-Cun Wang, Qing-Qing Yuan, Jing Xia, Li-Fang Cao, Kui Liu

**Affiliations:** Department of Respiratory and Critical Care Medicine, The Affiliated Bozhou Hospital of Anhui Medical University, Bozhou, Anhui, China

**Keywords:** ALI, asthma, all-cause mortality, cause-specific mortality, competing risk model, NHANES

## Abstract

**Background:**

The advanced lung cancer inflammation index (ALI), which reflects both inflammation and nutritional status, has an uncertain role in predicting outcomes for asthma patients. This study aimed to evaluate the association between ALI and mortality from all causes, as well as specific causes including cardiovascular disease (CVD) and cancer-related mortality, among individuals with asthma.

**Methods:**

We analyzed data from 4,829 asthma patients who participated in the U.S. National Health and Nutrition Examination Survey (NHANES) from 1999 to 2018. Cox proportional hazards models were used to assess the relationship between ALI and both all-cause and cause-specific mortality, adjusting for demographic and clinical variables. Additionally, restricted cubic spline models were applied to explore potential nonlinear trends, while segmented Cox models were used to identify threshold effects. A competing risk model further examined the independent association of ALI with CVD mortality.

**Results:**

Over a median follow-up of 7.83 years, a total of 582 deaths from all causes, 151 cardiovascular-related deaths, and 125 cancer-related deaths were recorded. An L-shaped association was observed between ALI and both all-cause and CVD mortality, with thresholds identified at 82.02 for all-cause mortality and 58.40 for CVD mortality. Compared to the lowest quartile of ALI (Q1), patients in the highest quartile (Q4) had a 49% lower risk of all-cause mortality (hazard ratio [HR] 0.51, 95% confidence interval [CI] 0.40–0.66) and a 51% reduction in CVD mortality (HR 0.49, 95% CI 0.29–0.83). This protective effect was further confirmed by the competing risk model. No significant association between ALI and cancer mortality was observed (HR 1.01, 95% CI 0.98–1.03).

**Conclusion:**

ALI was significantly and inversely associated with all-cause and CVD mortality in asthma patients, particularly when ALI values were below 82.02 and 58.40, respectively, where the risk of mortality was substantially lower. These findings suggest that ALI may have clinical utility in assessing prognosis for asthma patients, especially in terms of cardiovascular risk evaluation.

## Introduction

Asthma is a prevalent chronic respiratory disease that affects millions worldwide (1). Patients often experience symptoms such as wheezing, breathlessness, chest tightness, and coughing, which significantly impact their quality of life (2). Despite a range of treatment options, asthma continues to lead to frequent exacerbations, higher hospitalization rates, and a notable decline in quality of life. Epidemiological studies have demonstrated a strong link between asthma and mortality from various causes, particularly cardiovascular and respiratory conditions (3, 4). In addition, cardiovascular comorbidities such as diabetes mellitus, chronic kidney disease, and stroke may contribute to asthma-related mortality by exacerbating systemic inflammation and metabolic dysregulation. Therefore, early identification of high-risk asthma patients through reliable prognostic biomarkers and targeted interventions is essential for improving outcomes (5).

Asthma involves multiple mechanisms, including immune system dysregulation, airway hyperreactivity, and remodeling (6). Inflammatory processes in the airways, marked by the presence of eosinophils, basophils, and T lymphocytes, indicate abnormal activation of both innate and adaptive immunity (7). Cytokines such as IL-4, IL-5, and IL-13, released by these immune cells, contribute to airway pathology, resulting in airflow limitation (8). Recent evidence suggests that asthma can also provoke systemic immune-inflammatory responses (9). This has prompted investigations into the role of systemic immune markers in predicting asthma outcomes. For instance, the neutrophil-to-lymphocyte ratio (NLR) and platelet-to-lymphocyte ratio (PLR), which reflect immune status, have been associated with asthma control and the risk of exacerbations (10, 11). These findings underscore the need for further research into comprehensive markers of immune inflammation in asthma.

In this context, the Advanced Lung Cancer Inflammation Index (ALI) has emerged as a novel marker of inflammation, attracting considerable attention from researchers. ALI captures both nutritional status and systemic inflammation by combining body mass index (BMI), serum albumin, and NLR (11). As an integrated marker of nutrition and inflammation, ALI provides a broader assessment of a patient’s overall condition compared to individual markers, making it particularly useful in prognostic evaluations across various diseases. Initially studied in non-small cell lung cancer, ALI showed a strong ability to predict survival outcomes (11, 12). Its use has expanded to other conditions, including gastric cancer (13), colorectal cancer (14), hepatocellular carcinoma (15), as well as non-cancerous diseases like hypertension (16) and coronary artery disease (17). Recent studies by Trimarchi et al. (18) have demonstrated that ALI exhibits a strong predictive ability for all-cause mortality in patients with ST-segment elevation myocardial infarction (STEMI) undergoing primary percutaneous coronary intervention (PCI). This finding further supports the broad applicability of ALI as a global prognostic evaluation tool. Moreover, in patients with diabetes, studies have shown a significant association between ALI and both all-cause mortality and cardiovascular mortality, highlighting its important clinical value in assessing inflammatory status and nutritional levels (19). Collectively, these observations confirm ALI’s role as a comprehensive indicator of systemic inflammation and nutritional status, with wide-ranging clinical applications.

However, despite asthma being a chronic inflammatory airway disease characterized by complex immune responses and systemic effects, the prognostic value of ALI in asthma remains underexplored. Given the systemic inflammation and nutritional changes in asthma patients, investigating ALI’s potential as a prognostic tool is clinically significant. Therefore, this study aims to systematically assess the association between ALI and all-cause and cause-specific mortality in asthma patients, evaluating its promise as a novel prognostic tool and proposing strategies for improving long-term asthma management.

To evaluate the predictive potential of ALI in asthma, this study utilized data from 4,829 asthma patients in the NHANES 1999–2018 dataset. We examined the link between ALI and the risks of all-cause, cardiovascular disease (CVD), and cancer-related mortality, identified potential nonlinear patterns and thresholds, and performed subgroup analyses. Through this comprehensive assessment, we aim to offer epidemiological support for the use of ALI in asthma prognosis, providing insights into risk classification and tailored therapeutic approaches for asthma management.

## Methods and study population

This research utilized data from the National Health and Nutrition Examination Survey (NHANES), which was carried out in the U.S. between 1999 and 2018. Conducted by the Centers for Disease Control and Prevention (CDC), NHANES employed a complex, stratified, multistage probability sampling technique to gather health and nutrition data from a wide demographic, including adults and children (20). Ethical approval for the NHANES procedures was obtained from the National Center for Health Statistics (NCHS) Ethics Review Board, with informed consent secured from all participants prior to the survey (21).

**Figure 1:**
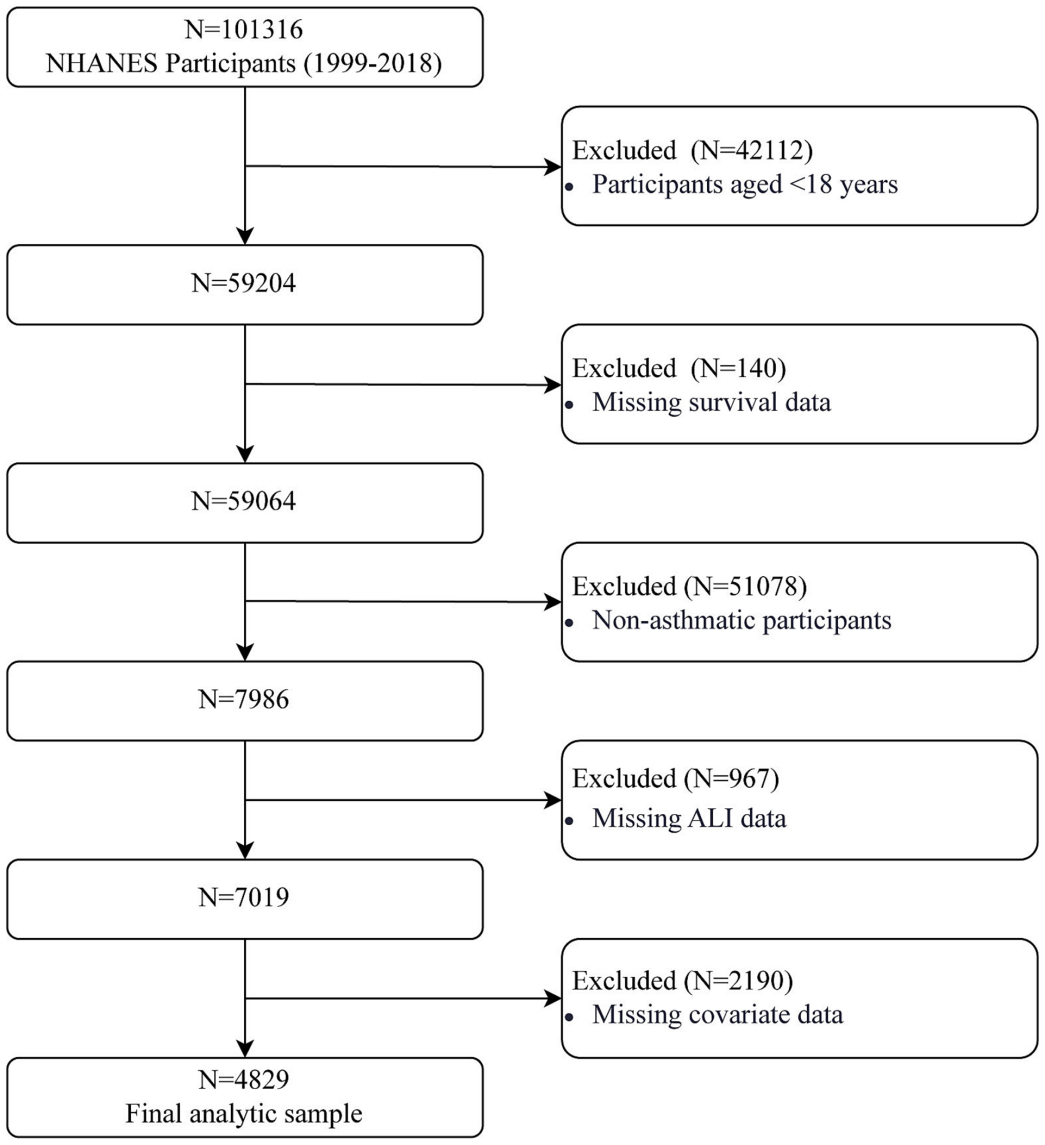
Flow chart of study participants.

A total of *N* = 101,316 participants from NHANES (1999–2018) were initially screened. After excluding participants aged <18 years (*N* = 42,112), *N* = 59,204 adult participants remained. We further excluded participants with missing survival data (*N* = 140), non-asthmatic participants (*N* = 51,078), those with missing ALI data (*N* = 967), and those with missing covariate data (*N* = 2,190). Finally, *N* = 4,829 eligible asthma patients were included in the final analytic sample. The selection process is depicted in Figure 1.

### Definitions and measurements of asthma and ALI

Asthma diagnosis was based on participants’ self-reported responses to the question “Have you ever been diagnosed with asthma by a doctor?” Those who answered “yes” were classified as asthma cases.

ALI was used to evaluate participants’ exposure, incorporating BMI, serum albumin (Alb), and NLR to assess nutritional and inflammatory status. The ALI formula is defined as BMI (kg/m^2^) × Alb (g/dL) / NLR, where NLR is calculated by dividing the neutrophil count by the lymphocyte count (22). Participants were divided into four quartiles according to ALI values: Q1 (lowest), Q2, Q3, and Q4 (highest), to investigate the impact of different ALI levels on all-cause and cause-specific mortality in asthma patients.

### Mortality data

The connection between NHANES and the National Death Index (NDI) facilitated access to mortality records, with follow-up concluding on December 31, 2019 (23). The NDI used probabilistic matching to determine the date and cause of death, which were classified under the International Classification of Diseases, 10th Revision (ICD-10) (24). This study examined mortality from all causes, including cardiovascular disease (CVD) and cancer. CVD deaths were identified using ICD-10 codes I00-I09, I11, I13, and I20-I51, while cancer deaths corresponded to codes C00-C97. The follow-up period began with participants’ initial examinations at the NHANES Mobile Examination Center (MEC) and continued until death, loss to follow-up, or the study’s endpoint on December 31, 2019.

### Definitions of variables of interest

This study adjusted for multiple covariates to reduce the impact of confounding factors. These covariates included demographic characteristics such as age, gender, and race (Non-Hispanic White, Non-Hispanic Black, Mexican American, and others); socioeconomic factors like education level, family income to poverty ratio (PIR), and marital status (married/partnered or single); lifestyle factors, including smoking status (current, former, or never) and alcohol use (yes/no); as well as health conditions such as hypertension, diabetes, cancer, and atherosclerotic cardiovascular disease (ASCVD), and chronic bronchitis,all based on self-reported data from questionnaires. Peripheral blood eosinophils were also included as a covariate, given their established role in asthma progression and prognosis. Additional details on these covariates are provided in the Supplementary methods.

### Statistical analyses

In accordance with the NHANES analysis guidelines, this study considered the complex sampling design and sampling weights. MEC weights were used for weighted analysis. We utilized the four-year MEC weight set (WTMEC4YR) because we included NHANES data from 1999 to 2000 and 2001–2002. For NHANES data from 2003 to 2018, two-year MEC weights (WTMEC2YR) were employed. The sampling weights for 1999–2018 were calculated as follows: weights for 1999–2002 were 2/10 × WTMEC4YR, while weights for other years were 1/10 × WTMEC2YR. Continuous variables were summarized using the mean and standard deviation, or the median and interquartile range, depending on the data distribution. Categorical variables were presented as counts and percentages. For group comparisons of continuous variables, one-way ANOVA or Kruskal-Wallis tests were used, while categorical variables were analyzed with Chi-square tests or Fisher’s exact test when expected frequencies were below 5. Missing data were handled using multiple imputation with chained equations (MICE), generating 5 imputed datasets. Predictive mean matching was employed for continuous variables, while binary logistic regression was applied for categorical variables (25). The imputation model included covariates, exposure variables, outcome variables, and other relevant predictors. Pooled results from the imputed datasets were used in all subsequent analyses following Rubin’s rules.

Cox proportional hazards models were used to assess the relationship between ALI and the risks of all-cause and cause-specific mortality (including CVD and cancer) in asthma patients. ALI was considered both as a continuous variable (with values divided by 10 to estimate risk per unit increase) and a categorical variable based on quartiles, with Q1 as the reference. Hazard ratios (HR) and 95% confidence intervals (CI) were calculated for each model. Three models were constructed: an unadjusted model; Model 1, adjusted for gender, age, race, education level, marital status, and PIR; and Model 2, which additionally adjusted for smoking status, alcohol use, ASCVD, hypertension, diabetes, cancer, eosinophils, and chronic bronchitis.

The Kaplan–Meier method was employed to estimate survival functions by ALI group, and group differences were evaluated using the log-rank test.

First, a restricted cubic spline (RCS) model was used with knots placed at the 5th, 35th, 65th, and 95th percentiles of the ALI distribution to evaluate the nonlinear relationship between ALI and the risks of all-cause and CVD mortality among asthma patients. The median ALI value served as the reference, and adjusted HR curves were plotted to visualize the association.

Next, segmented Cox proportional hazards models were used to further assess the relationship between ALI levels and mortality risks, controlling for confounders in Model 2. The likelihood ratio test was used to identify threshold effects and potential inflection points.

Stratified analyses were performed by gender, age (<60 years vs. ≥60 years), smoking status (current, former, never), alcohol use, ASCVD, hypertension, diabetes, and cancer and chronic bronchitis to evaluate variations in ALI’s impact on all-cause and CVD mortality.

In this study, participants with ASCVD, cancer, and diabetes were sequentially excluded for multivariate analyses to minimize the impact of these conditions on the results. To assess the association between ALI and CVD mortality, the Fine and Gray competing risks model was applied, treating non-cardiovascular deaths as competing events. Additionally, to ensure the robustness of the findings, extreme ALI values (mean ± 3 SD) were excluded to reduce their potential influence on the analysis. Finally, an additional analysis excluding missing values was planned to ensure consistency with the results obtained using multiple imputation.

Data analysis was performed with R software (version 4.2.1; R Foundation for Statistical Computing), the R survey package (version 4.1–1), and Free Statistics software (version 2.0; Beijing Free Clinical Medical Technology Co., Ltd.) (26). For interaction tests, Bonferroni correction was applied for multiple comparisons, setting a significance level at *α* = 0.0056 (0.05/9). For all other analyses, a two-sided *p*-value below 0.05 was regarded as statistically significant.

## Results

### Baseline characteristics

A total of 4,829 participants were included in the study, with a mean age of 47.06 ± 17.77 years, and 40.77% of the participants were male. The median ALI was 62.89, with an interquartile range of 45.18 to 86.40. Table 1 presents the baseline characteristics across ALI quartiles. Significant differences (*p* < 0.05) were found among the quartiles in variables such as age, race, smoking status, PIR, eosinophils, hypertension, chronic bronchitis, and cancer. Participants in the higher ALI quartiles (Q2, Q3, and Q4), compared to the lowest quartile (Q1), tended to have a younger average age, higher eosinophils, a larger proportion of non-Hispanic Black individuals, and lower incidences of chronic bronchitis and cancer.

**Table 1:** Baseline characteristics of participants.

Characteristics	Total	ALI				*p-*value
		Quantile 1 (2.89–45.18)	Quantile 2 (45.18–62.89)	Quantile 3 (62.98–86.36)	Quantile 4 (86.36–941.74)	
Participants, n	4,829	1,207	1,207	1,207	1,208	
Gender, n (%)						0.814
Male	1995 (40.77)	503 (37.35)	489 (39.77)	510 (43.44)	493 (42.59)	
Female	2,834 (59.23)	704 (62.65)	718 (60.23)	697 (56.56)	715 (57.41)	
Age(years)	47.06 ± 17.77	49.52 ± 19.86	46.69 ± 17.15	46.31 ± 17.31	45.74 ± 16.35	<0.001
Race, n (%)						<0.001
Non-Hispanic White	2,358 (70.88)	710 (77.26)	632 (73.95)	586 (71.45)	430 (59.34)	
Non-Hispanic Black	1,143 (12.13)	178 (7.06)	228 (9.50)	267 (10.85)	470 (22.58)	
Mexican American	471 (4.81)	100 (4.15)	114 (4.42)	142 (5.51)	115 (5.16)	
Other	857 (12.18)	219 (11.53)	233 (12.13)	212 (12.20)	193 (12.92)	
Education level, n (%)						0.169
<9	375 (4.19)	99 (4.11)	97 (4.12)	83 (3.77)	96 (4.85)	
9–12	1749 (32.44)	453 (35.03)	407 (28.49)	428 (31.16)	461 (35.83)	
>12	2,705 (63.37)	655 (60.86)	703 (67.39)	696 (65.07)	651 (59.29)	
Marital Status, n (%)						0.372
Married or living with partners	2,625 (58.85)	642 (56.12)	680 (61.18)	659 (59.44)	644 (58.42)	
Living alone	2,204 (41.15)	565 (43.88)	527 (38.82)	548 (40.56)	564 (41.58)	
Smoking status, n (%)						< 0.001
Current	1,152 (23.34)	313 (27.22)	312 (23.92)	265 (20.66)	262 (21.56)	
Former	1,220 (25.42)	341 (28.01)	265 (21.86)	299 (26.07)	315 (26.01)	
Never	2,457 (51.23)	553 (44.77)	630 (54.22)	643 (53.27)	631 (52.44)	
Alcohol Use, n (%)						0.114
Yes	3,623 (79.19)	908 (79.92)	934 (81.35)	885 (77.00)	896 (78.42)	
No	1,206 (20.81)	299 (20.08)	273 (18.65)	322 (23.00)	312 (21.58)	
Hypertension, n (%)						< 0.001
Yes	1965 (35.77)	472 (34.57)	449 (32.38)	472 (35.18)	572 (41.93)	
No	2,864 (64.23)	735 (65.43)	758 (67.62)	735 (64.82)	636 (58.07)	
Diabetes, n (%)						0.072
Yes	764 (11.27)	168 (9.29)	183 (10.87)	201 (11.76)	212 (13.36)	
No	4,065 (88.73)	1,039 (90.71)	1,024 (89.13)	1,006 (88.24)	996 (86.64)	
Cancer, n (%)						0.019
Yes	513 (11.08)	156 (11.74)	126 (12.57)	120 (10.24)	111 (9.58)	
No	4,316 (88.92)	1,051 (88.24)	1,081 (87.43)	1,087 (89.76)	1,097 (90.42)	
ASCVD, n (%)						0.126
Yes	649 (10.77)	185 (12.14)	163 (10.50)	152 (10.22)	149 (10.23)	
No	4,180 (89.23)	1,022 (87.86)	1,044 (89.50)	1,055 (89.78)	1,059 (89.77)	
Chronic bronchitis,n (%)						<0.001
Yes	988 (19.96)	293 (23.64)	218 (19.12)	221 (17.46)	256 (19.83)	
No	3,841 (80.04)	914 (76.36)	989 (80.88)	986 (82.52)	952 (80.17)	
PIR	1.89 (1.01, 3.88)	1.84 (1.02, 3.87)	2.03 (1.02, 4.13)	2.02 (1.02, 4.02)	1.75 (0.91, 3.56)	0.009
Eosinophils, ×10^9^/L	30.68 ± 8.09	27.13 ± 6.22	29.52 ± 7.25	31.51 ± 7.60	34.55 ± 9.12	<0.001
All-cause mortality n (%)						<0.001
Alive	4,247 (91.21)	963 (84.62)	1,078 (92.51)	1,086 (92.88)	1,120 (95.01)	
Death	582 (8.79)	244 (15.38)	129 (7.49)	121 (7.12)	88 (4.99)	
CVD mortality, n (%)						<0.001
Alive	4,678 (97.75)	1,142 (96.06)	1,170 (97.95)	1,178 (98.19)	1,188 (98.90)	
Death	151 (2.25)	65 (3.94)	37 (2.05)	29 (1.81)	20 (1.10)	
Cancer mortality, n (%)						0.057
Alive	4,704 (98.30)	1,164 (97.13)	1,178 (98.63)	1,176 (98.45)	1,186 (99.02)	
Death	125 (1.70)	43 (2.87)	29 (1.37)	31 (1.55)	22 (0.98)	
ALI	62.89(45.18,86.40)	34.48(26.73,40.52)	54.53(49.82,58.54)	72.83(67.58,78.72)	109.71(96.03,132.7)	<0.001

Data presented as unweighted numbers (weighted percentage) for categorical variables and mean (standard error) for continuous variables. This table is based on the complete-case sample (*n* = 4,829), while subsequent analyses used multiple imputation with a total sample size of 7019. Variables with missing data and their respective missing proportions: Education level: 8.82%, Marital Status: 5.04%, Smoking status: 6.64%, Alcohol Use: 20.26%, Hypertension: 0.17%, Diabetes: 2.34%, Cancer: 8.88%, ASCVD: 8.73%, Chronic bronchitis: 9.13%, PIR: 7.66%. ALI, advanced lung cancer inflammation index; CVD, Cardiovascular Disease; PIR, Ratio of family income to poverty; ASCVD, Atherosclerotic Cardiovascular Disease.

As of December 31, 2019, the median follow-up period was 7.83 years. During this time, 582 participants (8.79%) died from all causes, 151 (2.25%) from CVD, and 125 (1.70%) from cancer. To evaluate the influence of baseline characteristics on mortality risk, univariate Cox regression analysis was conducted. The results indicated that ALI was significantly associated with all-cause and CVD mortality (*p* < 0.001), but not with cancer mortality (*p* = 0.778). In the all-cause mortality analysis, all variables except gender showed statistical significance (*p* < 0.05). For CVD mortality, all variables except gender, race, smoking status and alcohol use reached statistical significance (*p* < 0.05). In the cancer mortality analysis, variables such as gender, race, marital status, alcohol use, and eosinophils did not reach statistical significance (*p* > 0.05). Detailed results of the univariate Cox regression analyses are provided in Supplementary Tables S1–S3.

### Associations between ALI and mortality

Table 2 presents the findings from the Cox proportional hazards regression analyses on the associations between ALI and mortality among asthma patients. After adjusting for confounders in Model 2, ALI showed a significant inverse relationship with all-cause and CVD mortality, but no significant association with cancer mortality.

**Table 2:** Multivariate analysis of the association between ALI and all-cause, CVD and cancer mortality of asthma.

ALI	Events	Crude model		Model 1		Model 2	
		HR, 95%CI	*p-*value	HR, 95%CI	*p-*value	HR, 95%CI	*p-*value
All-cause mortality							
Per10Uincrement	881(12.6)	0.92(0.88–0.95)	< 0.001	0.96(0.92–0.99)	0.014	0.96(0.92–0.99)	0.015
Quantile 1	359(20.5)	1(Ref)		1(Ref)		1(Ref)	
Quantile 2	200(11.4)	0.52(0.42 ~ 0.63)	<0.001	0.67(0.54 ~ 0.82)	<0.001	0.67(0.55 ~ 0.82)	<0.001
Quantile 3	183(10.4)	0.51(0.41 ~ 0.64)	<0.001	0.65(0.51 ~ 0.82)	<0.001	0.65(0.50 ~ 0.84)	0.001
Quantile 4	139(7.9)	0.38(0.30 ~ 0.47)	<0.001	0.52(0.41 ~ 0.66)	<0.001	0.51(0.40 ~ 0.66)	<0.001
*P* for trend			<0.001		<0.001		<0.001
CVD mortality							
Per10Uincrement	220(3.1)	0.89(0.83 ~ 0.96)	0.001	0.94(0.89 ~ 0.99)	0.033	0.93(0.88 ~ 0.99)	0.027
Quantile 1	85(4.8)	1(Ref)		1(Ref)		1(Ref)	
Quantile 2	54(3.1)	0.63(0.43 ~ 0.93)	0.019	0.87(0.58 ~ 1.29)	0.477	0.86(0.58 ~ 1.28)	0.454
Quantile 3	49(2.8)	0.68(0.42 ~ 1.09)	0.11	0.93(0.57 ~ 1.51)	0.769	0.90(0.55 ~ 1.48)	0.680
Quantile 4	32(1.8)	0.36(0.22 ~ 0.58)	<0.001	0.52(0.31 ~ 0.87)	0.013	0.49(0.29 ~ 0.83)	0.008
*P* for trend			<0.001		0.064		0.037
Cancer mortality							
Per10Uincrement	178(2.5)	0.99(0.90,1.08)	0.778	1.01(0.98,1.03)	0.691	1.01(0.98,1.03)	0.643
Quantile 1	60(3.4)	1(Ref)		1(Ref)		1(Ref)	
Quantile 2	42(2.4)	0.57(0.36 ~ 0.92)	0.020	0.74(0.45 ~ 1.20)	0.221	0.77(0.48 ~ 1.24)	0.277
Quantile 3	43(2.5)	0.57(0.35 ~ 0.94)	0.027	0.75(0.45 ~ 1.25)	0.273	0.76(0.44 ~ 1.29)	0.306
Quantile 4	33(1.9)	0.51(0.29 ~ 0.90)	0.021	0.71(0.39 ~ 1.28)	0.254	0.78(0.43 ~ 1.41)	0.409
*P* for trend			0.018		0.226		0.335

Crude Model: no covariates were adjusted; Model 1: Adjusted for gender, age, race, education level, marital status, PIR; Model 2 was adjusted for model 1+ smoking status, alcohol use, ASCVD, hypertension, diabetes, cancer, eosinophils, chronic bronchitis. Analyses were based on 5 imputed datasets using multiple imputation to address missing data. ALI advanced lung cancer inflammation index; CVD, Cardiovascular Disease; PIR, Ratio of family income to poverty; ASCVD, Atherosclerotic Cardiovascular Disease; Ref, Reference.

For all-cause mortality, each 10-unit increase in ALI was associated with a 4% reduction in the risk of death (HR 0.96, 95% CI 0.92–0.99, *p* = 0.015). When ALI levels were categorized into quartiles, participants in the highest quartile (Q4) had a 49% lower risk of all-cause mortality than those in the lowest quartile (Q1; HR 0.51, 95% CI 0.40–0.66, *p* < 0.001). The trend test was statistically significant (p for trend <0.001).

Similarly, ALI levels were significantly and inversely associated with CVD mortality. A 10-unit increase in ALI was linked to a 7% decrease in the risk of cardiovascular death (HR 0.93, 95% CI 0.88–0.99, *p* = 0.027). In the categorical analysis, participants in Q4 experienced a 51% lower risk of cardiovascular death compared to Q1 (HR 0.49, 95% CI 0.29–0.83, *p* = 0.008). The trend test also showed statistical significance (p for trend = 0.037).

However, ALI did not exhibit a significant association with cancer mortality. A 10-unit increase in ALI yielded an HR for cancer mortality of 1.01 (95% CI 0.98–1.03, *p* = 0.643). In the categorical analysis, the HR for Q4 versus Q1 was 0.78 (95% CI 0.43–1.41, *p* = 0.409). The trend test did not show statistical significance (p for trend = 0.335).

### Kaplan–Meier survival analysis

**Figure 2:**
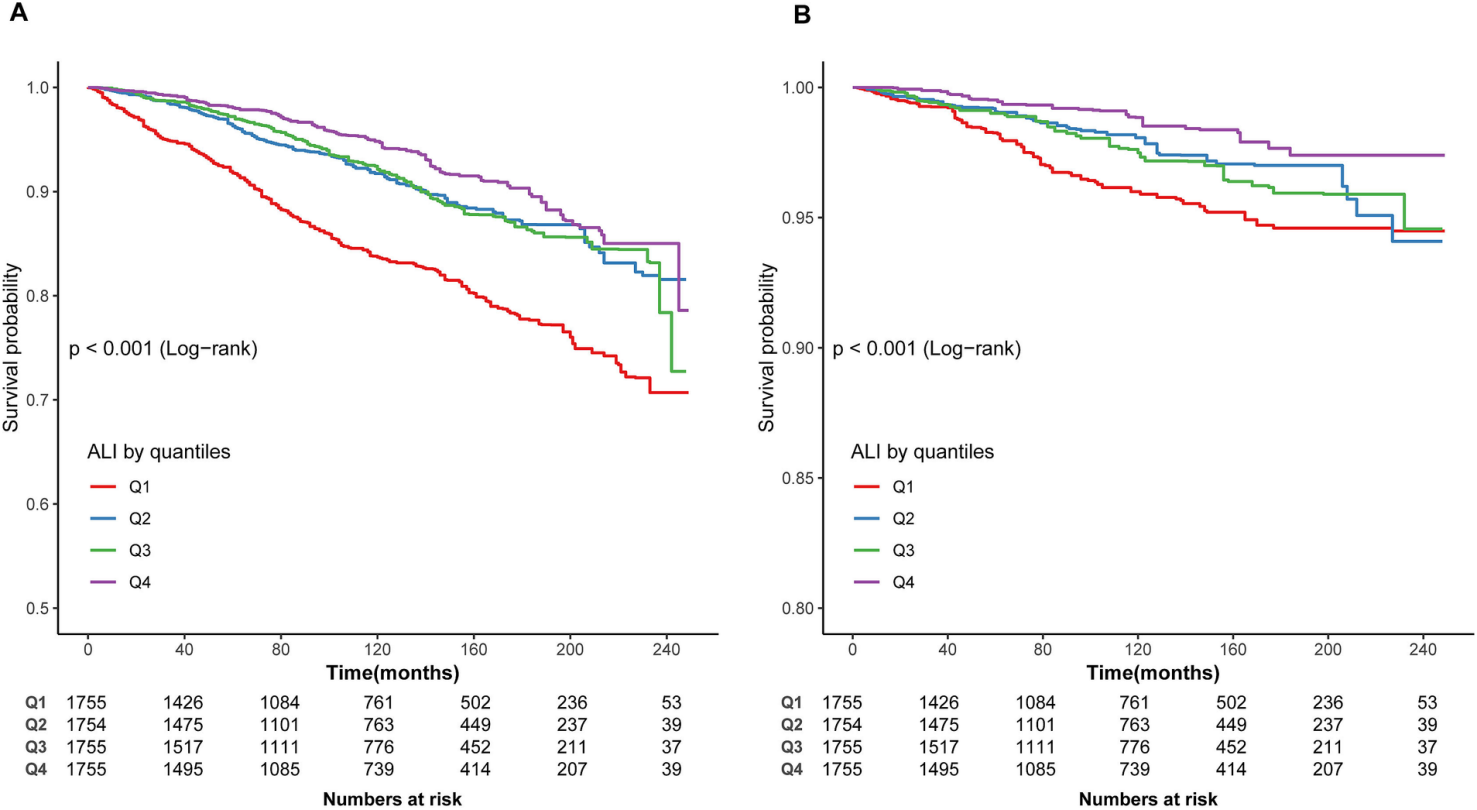
Kaplan–Meier survival curve. **(A)** Kaplan–Meier survival curve for all-cause mortality. **(B)** Kaplan–Meier survival curve for CVD mortality. In the Kaplan–Meier curves, the population is stratified into four groups (Q1, Q2, Q3, Q4) based on the quartiles of ALI, and statistical analysis is conducted using the log-rank test.

As shown in Figures 2A,B, Kaplan–Meier analysis identified significant associations between ALI levels and both all-cause (log-rank *p* < 0.001) and CVD mortality (log-rank *p* < 0.001) in asthma patients during a median follow-up of 7.83 years. Participants in the highest ALI quartile (Q4) exhibited a higher cumulative survival rate than those in the lowest quartile (Q1). Additionally, the association between ALI levels and cancer mortality was assessed (Supplementary Figure S1), with the log-rank test showing no significant differences in cancer mortality across ALI quartiles (*p* = 0.11).

### Nonlinear relationship between ALI and mortality and dose–response analysis

**Figure 3:**
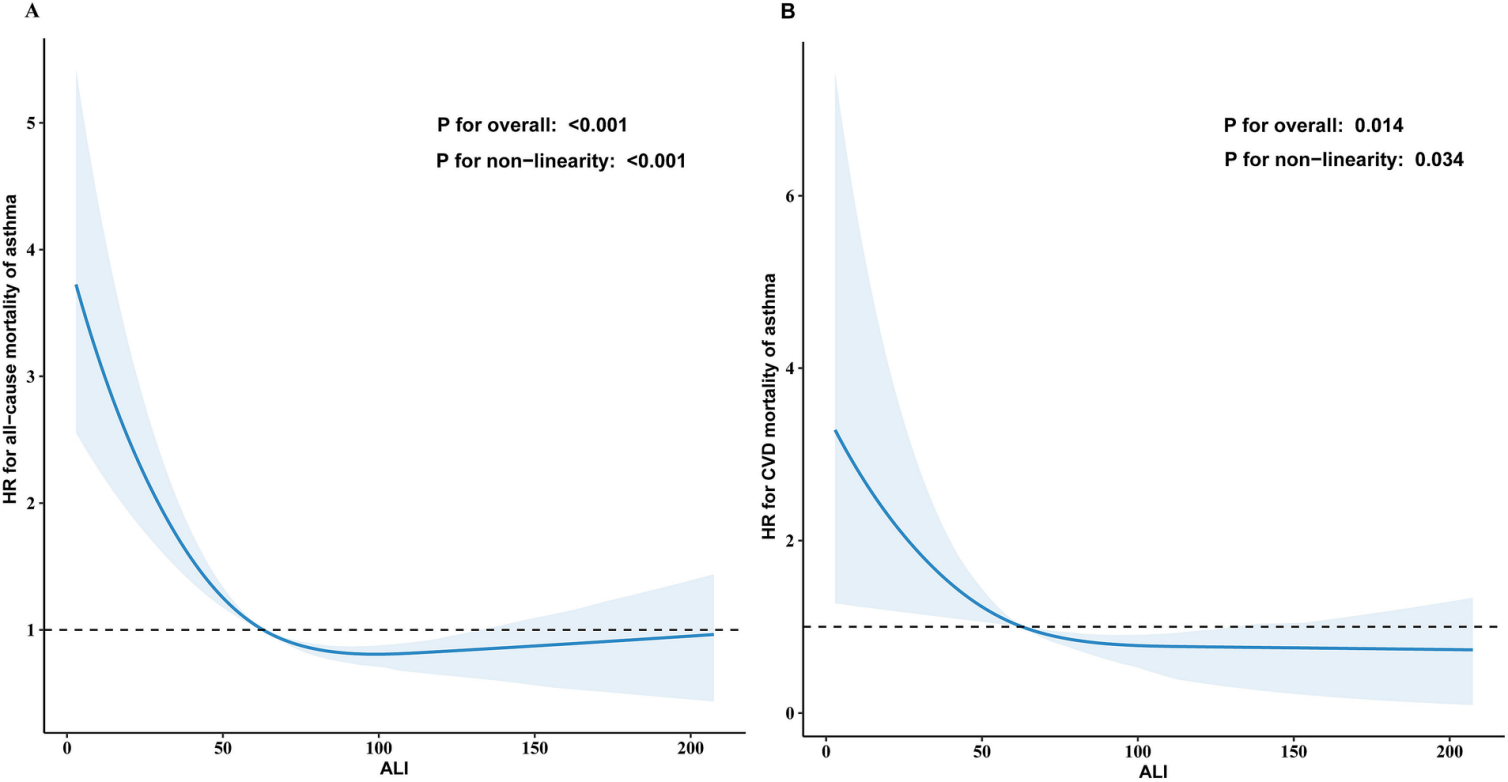
The association of ALI with all-cause **(A)** and CVD mortality **(B)** among asthma visualized by restricted cubic spline. The solid blue line represents the smooth curve fit between the variables, and the light blue shaded areas represent the 95% confidence interval from the fit. The hazard ratios were adjusted for gender, age, race, education level, marital status, PIR, smoking status, alcohol use, ASCVD, hypertension, diabetes, cancer, eosinophils and chronic bronchitis. Only 99% of the data is shown.

In the multivariable Cox regression analysis, the relationship between ALI and both all-cause mortality and CVD mortality was found to be non-linear. RCS analysis revealed an L-shaped relationship between ALI and all-cause as well as CVD mortality (Figures 3A,B). A threshold effect analysis identified a turning point for all-cause mortality at an ALI of 82.02. Below this threshold, each 1-unit increase in ALI corresponded to a 1% reduction in all-cause mortality risk (HR 0.99, 95% CI 0.98–0.99, *p* < 0.001). When ALI was ≥82.02, the mortality risk increased significantly (HR 1.00, 95% CI 0.99–1.01, *p* = 0.104).

For CVD mortality, the turning point was identified at an ALI of 58.40. Below this threshold, each 1-unit increase in ALI correlated with a 3% reduction in CVD mortality risk (HR 0.97, 95% CI 0.94–0.99, *p* = 0.002), while no significant association was found for ALI ≥58.40 (HR 0.99, 95% CI 0.98–1.01, *p* = 0.320; Table 3). No non-linear relationship was found for cancer mortality (Supplementary Figure S2).

**Table 3:** Threshold effect analysis of ALI on all-cause and CVD mortality in participants with asthma.

ALI	Adjusted HR (95% CI)	*p*-value
All-cause mortality		
ALI < 82.02	0.99 (0.98–0.99)	<0.001
ALI ≥ 82.02	1.00 (0.99–1.01)	0.104
Likelihood Ratio test		<0.001
CVD mortality		
ALI < 58.40	0.97 (0.94–0.99)	0.002
ALI ≥ 58.40	0.99(0.98–1.01)	0.320
Likelihood Ratio test		0.018

Adjusted for gender, age, race, education level, marital status, PIR, smoking status, alcohol use, ASCVD, hypertension, diabetes, cancer, eosinophils, chronic bronchitis.

### Subgroup analyses

**Figure 4:**
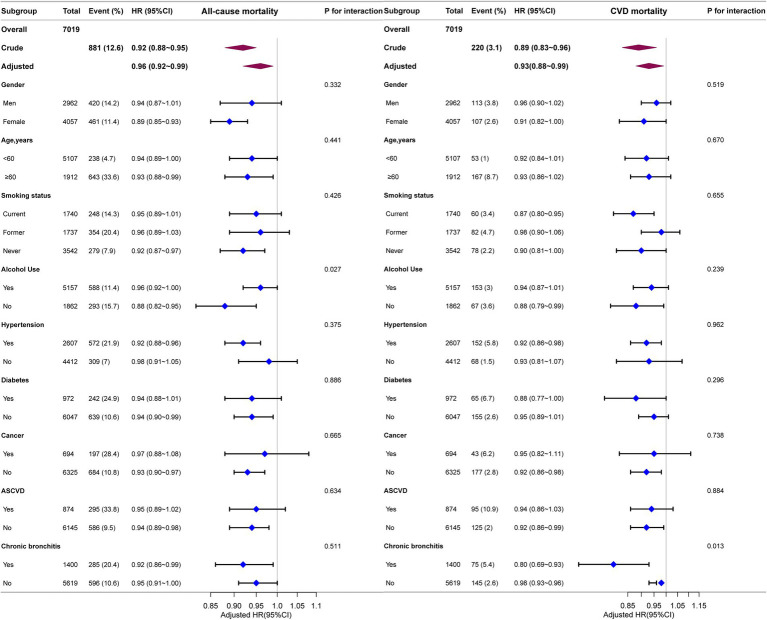
Forest plot for subgroup analysis of association between ALI and all-cause and CVD mortality. HRs were calculated using multivariate Cox regression models adjusted for the variables listed in the fully adjusted model except for the variable used for stratification.

We performed stratified subgroup analyses to comprehensively assess the association between ALI and the risks of all-cause and CVD mortality in asthma patients. The stratification factors included gender, age, smoking status, alcohol use, hypertension, diabetes, cancer, ASCVD and chronic bronchitis (Figure 4). The results demonstrated that the association between ALI and both all-cause and CVD mortality remained consistent across all subgroups (P for interaction >0.0056).

### Sensitivity analyses

In the sensitivity analyses, individuals with ASCVD (*n* = 6,145), cancer (*n* = 6,332), and diabetes (*n* = 6,052) were excluded, and the relationship between ALI and both all-cause and CVD mortality remained consistent with the main analysis (Supplementary Tables S4–S6). To further validate these results, a Fine and Gray model was utilized to account for competing causes of death, reaffirming the association between ALI and CVD mortality (Supplementary Table S7). Additionally, we conducted an analysis with a restricted ALI range (mean ± 3 SD), which included 6,958 participants. Even after adjusting for confounders, the association between ALI and both all-cause and CVD mortality remained statistically significant, while the link between ALI and cancer mortality did not achieve statistical significance in any model (Supplementary Table S8). Finally, we performed an additional analysis excluding missing values (n = 4,829), as presented in Supplementary Table S9. The results were consistent with those derived from the multivariable analysis using multiple imputation.

## Discussion

In this study of 4,829 asthma patients from the United States, we identified a significant negative correlation between ALI and both all-cause mortality and CVD mortality, while no significant association was observed with cancer-related mortality. Importantly, we detected a nonlinear L-shaped relationship between ALI and both all-cause and CVD mortality, with inflection points at 82.02 and 58.40, respectively. Below these thresholds, higher ALI values were associated with a substantial reduction in mortality risk. However, once ALI exceeded these inflection points, the associations became nonsignificant for both all-cause and CVD mortality. Subgroup and sensitivity analyses further reinforced the robustness of these findings. Our results underscore the potential utility of ALI as a prognostic biomarker for long-term outcomes in asthma patients, particularly in the context of personalized risk stratification and management. These findings suggest that ALI could be instrumental in identifying high-risk patients who may benefit from more tailored interventions, further supporting its relevance in the clinical management of asthma.

This study demonstrated that ALI was significantly inversely associated with all-cause and CVD mortality in asthma patients. This finding aligns with previous research, further supporting the potential of ALI as a prognostic marker. For example, oncology studies have demonstrated a significant association between higher ALI levels and prolonged survival in patients with non-small cell lung cancer (11, 12); in the context of cardiovascular diseases, lower ALI has been strongly linked to adverse outcomes in individuals with coronary artery disease (17, 27). Collectively, these studies validate the utility of ALI as a comprehensive indicator that reflects nutritional status, systemic inflammation, and immune function. In metabolic diseases, a complex nonlinear association has been observed between ALI and both all-cause and CVD mortality among patients with type 2 diabetes, consistent with our findings in asthma patients (19). Moreover, the use of ALI in chronic obstructive pulmonary disease (COPD) has been confirmed, showing significant associations with disease risk and all-cause mortality, with predictive performance superior to other nutritional markers (28). In asthma patients, a recent study by Fu et al. (29) based on NHANES data similarly reported that higher ALI levels were associated with reduced all-cause and respiratory disease mortality. However, Fu et al. identified the risk nadir for respiratory mortality at an ALI value of 109.13, whereas our study identified different inflection points for all-cause (82.02) and CVD mortality (58.40). These differences likely reflect variations in sample composition and analytical approaches but further emphasize the robustness of ALI as a predictive marker. Our study did not identify a significant association between ALI and cancer-related mortality in asthma patients, which may be due to the heterogeneity of ALI mechanisms across different disease contexts. The competing risk model further validated the independent inverse relationship between ALI and CVD mortality, highlighting its potential role in the long-term prognostic assessment of asthma patients. These results provide crucial scientific evidence supporting the use of ALI in the management of asthma and other chronic respiratory conditions.

This study supports the prognostic value of ALI in asthma patients, revealing a significant negative correlation between ALI and both all-cause mortality and CVD mortality. ALI, by integrating BMI, serum albumin, and NLR, provides a comprehensive reflection of a patient’s nutritional status, systemic inflammation, and immune dysregulation, all of which collectively influence patient outcomes (25–28). First, BMI, as a key indicator of nutritional status, is often associated with malnutrition when low, which weakens respiratory muscle function and increases the risk of respiratory failure and infections (29, 30). Moreover, malnutrition impairs immune function, further exacerbating airway and systemic inflammation, thereby elevating the risk of mortality (29). As a result, BMI not only reflects nutritional status but also impacts all-cause and CVD mortality by contributing to systemic inflammation (30). Studies have shown that both high and low BMI are closely associated with metabolic syndrome and the progression of atherosclerosis, which in turn increases the risk of CVD mortality (31, 32).

A decline in serum albumin levels indicates chronic inflammation and reduced antioxidant capacity, which is particularly evident in asthma patients (33, 34). Chronic low-grade inflammation not only exacerbates airway remodeling and oxidative stress but also increases the risk of cardiovascular complications (35, 36). Albumin possesses both antioxidant and anti-inflammatory characteristics, and is essential in sustaining normal physiological processes. (37). Studies have shown that low albumin levels are associated with increased all-cause mortality and may further elevate the risk of cardiovascular disease by enhancing oxidative stress and endothelial damage (38). Elevated serum albumin levels provide potential protective effects for asthma patients through its antioxidant and anti-inflammatory properties. Mechanistic studies confirm that high-level albumin relies on its free thiol group (Cys-34) to scavenge reactive oxygen species (ROS) and reactive nitrogen species (RNS), mitigating oxidative stress-induced damage in airway tissues and suppressing pro-inflammatory cytokines such as TNF-*α* and IL-6, thereby alleviating chronic inflammation and airway remodeling (39, 40). Epidemiological research further supports these findings. Each 1 g/L increase in albumin levels correlates with a 13% reduction in all-cause mortality among asthma patients, with significant improvements in lung function parameters (FVC and FEV1), suggesting that albumin may influence disease progression through modulation of oxidative stress and inflammatory responses (41, 42).

An elevated NLR reflects an enhanced neutrophil-driven inflammatory response accompanied by a decrease in lymphocytes, indicating immune dysregulation (43, 44). This immune imbalance in asthma patients not only exacerbates airway inflammation but also increases the risk of cardiovascular diseases through mechanisms such as accelerated atherosclerosis (45). Studies have demonstrated a significant association between elevated NLR and the risk of cardiovascular events, with higher NLR levels typically indicating more severe inflammation and increased CVD mortality (46–48). The competing risk model further supports the independent negative association between NLR and CVD mortality, highlighting the critical role of NLR within the ALI framework. In summary, ALI, through the combined influence of BMI, albumin, and NLR, reflects malnutrition, chronic inflammation, and immune dysregulation in asthma patients, which in turn impacts both all-cause and CVD mortality. Optimizing these parameters not only aids in improving patient outcomes but also underscores the potential of ALI in asthma risk assessment.

While our study investigated the relationship between ALI and mortality in asthma patients, it has some limitations that should be acknowledged. First, as a retrospective observational study, we cannot establish causality. Despite adjustments for multiple factors, unmeasured variables (such as medication use, diet, and physical activity) may have influenced the results. Second, this study utilized only baseline ALI data and lacked dynamic monitoring of ALI over time, preventing the evaluation of the long-term impact of ALI changes on mortality. Future research should incorporate multiple time-point measurements of ALI to better understand its prognostic role. Third, this study relied on self-reported physician-diagnosed asthma, which may introduce recall bias or misclassification. Participants might underreport or overreport their asthma status due to memory bias or differences in understanding the questions. Future research could incorporate objective diagnostic tools, such as pulmonary function tests or biomarkers, to improve diagnostic accuracy and enhance the reliability of the findings. Fourth, as this study was based on asthma patients in the United States, the generalizability of the results to other populations remains unclear. Future studies should aim to validate these findings in broader international cohorts. Lastly, the relatively short follow-up duration and the small sample size in some subgroups (e.g., the diabetes subgroup) may have limited the ability to identify differences between groups. Larger-scale studies are needed to confirm these findings.

## Conclusion

This study found a significant inverse association between ALI and both all-cause and CVD mortality in asthma patients, with no significant association observed for cancer-related mortality. Notably, an L-shaped nonlinear relationship was identified, with thresholds of 82.02 for all-cause mortality and 58.40 for CVD mortality, below which mortality risks declined sharply. These findings suggest that ALI is closely linked to long-term health outcomes in asthma patients, particularly in assessing cardiovascular risk.

## Data Availability

Publicly available datasets were analyzed in this study. This data can be found at: https://www.cdc.gov/nchs/nhanes/index.htm.
