# Supplementary material for: Association between advanced lung cancer inflammation index and all-cause and cause-specific mortality among asthma patients: a cohort study
Source: Front Nutr. 2025 Feb 6;12:1519271. doi: 10.3389/fnut.2025.1519271 (PMC11839423; doi:10.3389/fnut.2025.1519271)
Supplement: Supplementary file 1 [file Data_Sheet_1.pdf]

Supplementary Material

Supplementary Methods

Supplementary Table S1. Results of Univariate Cox Regression Analysis for Factors Associated with All-Cause Mortality in asthma**(N=7019)**

Supplementary Table S2. Results of Univariate Cox Regression Analysis for Factors Associated with CVD Mortality in asthma**(N=7019)**

Supplementary Table S3. Results of Univariate Cox Regression Analysis for Factors Associated with cancer Mortality in asthma**(N=7019)**

Supplementary Figure S1. Kaplan-Meier survival curve for cancer mortality

Supplementary Figure S2. Restricted cubic spline fitting for the association between ALI with cancer mortality.

Supplementary Table S4. Association of ALI with All-Cause, CVD, and Cancer Mortality in Asthma Patients from the NHANES Cohort, Excluding ASCVD Population **(n=6145)**

Supplementary Table S5. Association of ALI with All-Cause, CVD, and Cancer Mortality in Asthma Patients from the NHANES Cohort, Excluding cancer population **(n=6332)**

Supplementary Table S6. Association of ALI with All-Cause, CVD, and Cancer Mortality in Asthma Patients from the NHANES Cohort, Excluding diabetes population **(n=6052)**

Supplementary Table S7. Association between ALI and Cardiovascular Mortality using Fine & Gray Competing Risks Models

Supplementary Table S8. Multivariable Analysis of the Association Between ALI and All-Cause, Cardiovascular, and Cancer Mortality After Exclusion of Extreme Values **(n=6958)**

Supplementary Table S9. Multivariable Analysis of the Association Between ALI and All-Cause, Cardiovascular, and Cancer Mortality After Exclusion of Missing Values **(n=4829)**

Supplementary Methods

Description of covariate definitions

Gender, age, race/ethnicity, marital status, education level, and family income-to-poverty ratio (PIR) of participants were obtained from self-report at the time of the interview. Race/ethnicity was categorized as non-Hispanic White, non-Hispanic Black, Hispanic (Mexican American), and other. Marital status was classified as married or living with a partner, and living alone. Education level was categorized as < 9 years, 9-12 years, and > 12 years.

Alcohol using status was defined as follows: never (<12 drinks in a lifetime), previously (≥ 12 drinks in 1 year, no drinking in the last year, or no drinking in the last year but ≥ 12 drinks in a lifetime), and currently (≥ 1 drink/day on average in the past 12 months).

Smoking status was categorized as never (<100 cigarettes in their lifetime), previously (≥ 100 cigarettes in their lifetime, but not currently smoking), and currently (≥ 100 cigarettes in their lifetime, currently smoking occasionally or daily).

Hypertension was defined as meeting any of the following criteria: self-reported diagnosis by a physician or health professional, current use of antihypertensive medication, or average systolic/diastolic blood pressure ≥ 140/90 mmHg during the examination.

Diabetes was considered as a self-report of a physician or medical professional diagnosis, current use of antidiabetic medication, glycated hemoglobin (HbA1c) levels ≥ 6.5%, or fasting blood glucose (FBG) levels ≥ 126 mg/dl.

The diagnosis of ASCVD was based on self-reported questions from the MCQ section of the survey: “Have you ever been told you have coronary heart disease (CHD)?”; “Have you ever been told you have angina/angina pectoris?”; “Have you ever been told you had a heart attack?”; or “Have you ever been told you had a stroke?” A “yes” response to any of these questions was classified as ASCVD.

Cancer was defined as a self-report of ever being diagnosed with cancer or malignancy.

Chronic bronchitis was identified based on self-reported responses to the question: “Ever told you had chronic bronchitis?” Participants who answered “yes” were classified as having chronic bronchitis.

Peripheral blood cell counts (eosinophils, neutrophils, and lymphocytes) were obtained through routine complete blood count (CBC) testing in NHANES. Whole blood samples were analyzed in Mobile Examination Centers (MECs), and results were measured in duplicate, with the average values recorded.

Supplement Table S1. Results of Univariate Cox Regression Analysis for Factors Associated with All-Cause Mortality in asthma(N=7019)

Exposure	HR (95%CI)	*P-value*
**Age(years)**	1.09 (1.08,1.09)	**< 0.001**
**Gender, n (%)**		**0.971**
Male	1.0(ref)	
Female	0.99 (0.82,1.21)	
**Race, n (%)**		**0.012**
Non-Hispanic White	1.0(ref)	
Non-Hispanic Black	0.91 (0.73,1.12)	
Mexican American	0.49 (0.36,0.66)	
Other	0.74 (0.54,1.02)	
**Marital Status, n (%)**		**0.004**
Married	1.0(ref)	
Never married	1.35 (1.10,1.65)	
**PIR**	0.79 (0.75,0.83)	**< 0.001**
**Education level, n (%)**		**< 0.001**
<9	1.0(ref)	
9-12	0.38 (0.30,0.48)	
>12	0.22 (0.17,0.29)	
**Smoking status, n (%)**		**< 0.001**
Never	1.0(ref)	
Former	1.31 (1.08,1.59)	
Now	0.52 (0.42,0.66)	
**Alcohol Use, n (%)**		**0.001**
No	1.0(ref)	
Yes	0.74 (0.61,0.89)	
**Cancer, n (%)**		**< 0.001**
No	1.0(ref)	
Yes	3.13 (2.50,3.93)	
**Eosinophils**	1.92 (1.29,2.84)	**0.001**
**ASCVD, n (%)**		**< 0.001**
No	1.0(ref)	
Yes	5.59 (4.60,6.80)	
**Hypertension, n (%)**		**< 0.001**
No	1.0(ref)	
Yes	4.01 (3.30,4.87)	
**Diabetes, n (%)**		**< 0.001**
No	1.0(ref)	
Yes	3.99 (3.22,4.95)	
**Chronic bronchitis**		**< 0.001**
No	1.0(ref)	
Yes	2.11 (1.77,2.52)	
**ALI**	0.99 (0.98,0.99)	**< 0.001**

Abbreviations: ALI advanced lung cancer inflammation index; CVD Cardiovascular Disease; PIR Ratio of family income to poverty; ASCVD Atherosclerotic Cardiovascular Disease;

Supplement Table S2. Results of Univariate Cox Regression Analysis for Factors Associated with CVD Mortality in asthma(N=7019)

Exposure	HR (95%CI)	*P-value*
**Age(years)**	1.10 (1.09,1.12)	**< 0.001**
**Gender, n (%)**		**0.172**
Male	1.0(ref)	
Female	0.79 (0.56,1.11)	
**Race, n (%)**		**0.225**
Non-Hispanic White	1.0(ref)	
Non-Hispanic Black	1.26 (0.85,1.86)	
Mexican American	0.62 (0.32,1.19)	
Other	0.69 (0.37,1.27)	
**Marital Status, n (%)**		**0.005**
Married	1.0(ref)	
Never married	1.67 (1.17,2.38)	
**PIR**	0.76 (0.69,0.83)	**< 0.001**
**Education level, n (%)**		**< 0.001**
<9	1.0(ref)	
9-12	0.28 (0.18,0.42)	
>12	0.22 (0.15,0.32)	
**Smoking status, n (%)**		**0.066**
Never	1.0(ref)	
Former	1.47 (1.00,2.14)	
Now	0.75 (0.48,1.15)	
**Alcohol Use, n (%)**		**0.052**
No	1.0(ref)	
Yes	0.72 (0.51,1.00)	
**Cancer, n (%)**		**< 0.001**
No	1.0(ref)	
Yes	2.71 (1.83,4.01)	
**Eosinophils**	3.20 (1.87,5.45)	**< 0.001**
**ASCVD, n (%)**		**< 0.001**
No	1.0(ref)	
Yes	8.40 (6.10,11.57)	
**Hypertension, n (%)**		**< 0.001**
No	1.0(ref)	
Yes	5.00 (3.41,7.33)	
**Diabetes, n (%)**		**< 0.001**
No	1.0(ref)	
Yes	5.13 (3.46,7.62)	
**Chronic bronchitis, n (%)**		**< 0.001**
No	1.0(ref)	
Yes	2.25 (1.61,3.14)	
**ALI**	0.99 (0.98,1.00)	**0.001**

Abbreviations: ALI advanced lung cancer inflammation index; CVD Cardiovascular Disease; PIR Ratio of family income to poverty; ASCVD Atherosclerotic Cardiovascular Disease;

Supplement Table S3. Results of Univariate Cox Regression Analysis for Factors Associated with cancer Mortality in asthma(N=7019)

Exposure	HR (95%CI)	*P-value*
**Age(years)**	1.07 (1.07,1.10)	**< 0.001**
**Gender, n (%)**		**0.168**
Male	1.0(ref)	
Female	0.78 (0.54,1.11)	
**Race, n (%)**		**0.066**
Non-Hispanic White	1.0(ref)	
Non-Hispanic Black	0.93 (0.59,1.46)	
Mexican American	0.58 (0.33,1.02)	
Other	0.55 (0.26,1.14)	
**Marital Status, n (%)**		**0.523**
Married	1.0(ref)	
Never married	1.14 (0.77,1.69)	
**PIR**	0.82 (0.74,0.91)	**< 0.001**
**Education level, n (%)**		**< 0.001**
<9	1.0(ref)	
9-12	0.50 (0.28,0.91)	
>12	0.26 (0.14,0.48)	
**Smoking status, n (%)**		**< 0.001**
Never	1.0(ref)	
Former	1.56 (1.01,2.40)	
Now	0.38 (0.23,0.63)	
**Alcohol Use, n (%)**		**0.782**
No	1.0(ref)	
Yes	1.07 (0.68,1.68)	
**Cancer, n (%)**		**< 0.001**
No	1.0(ref)	
Yes	5.38 (3.46,8.37)	
**Eosinophils**	0.48 (0.17,1.32)	**0.154**
**ASCVD, n (%)**		**< 0.001**
No	1.0(ref)	
Yes	5.66 (3.58,8.95)	
**Hypertension, n (%)**		**< 0.001**
No	1.0(ref)	
Yes	2.74 (1.83,4.10)	
**Diabetes, n (%)**		**< 0.001**
No	1.0(ref)	
Yes	2.54 (1.63,3.97)	
**Chronic bronchitis, n (%)**		**0.040**
No	1.0(ref)	
Yes	1.48 (1.02,2.14)	
**ALI**	1.00 (0.99,1.01)	**0.778**

Abbreviations: ALI advanced lung cancer inflammation index; CVD Cardiovascular Disease; PIR Ratio of family income to poverty; ASCVD Atherosclerotic Cardiovascular Disease;

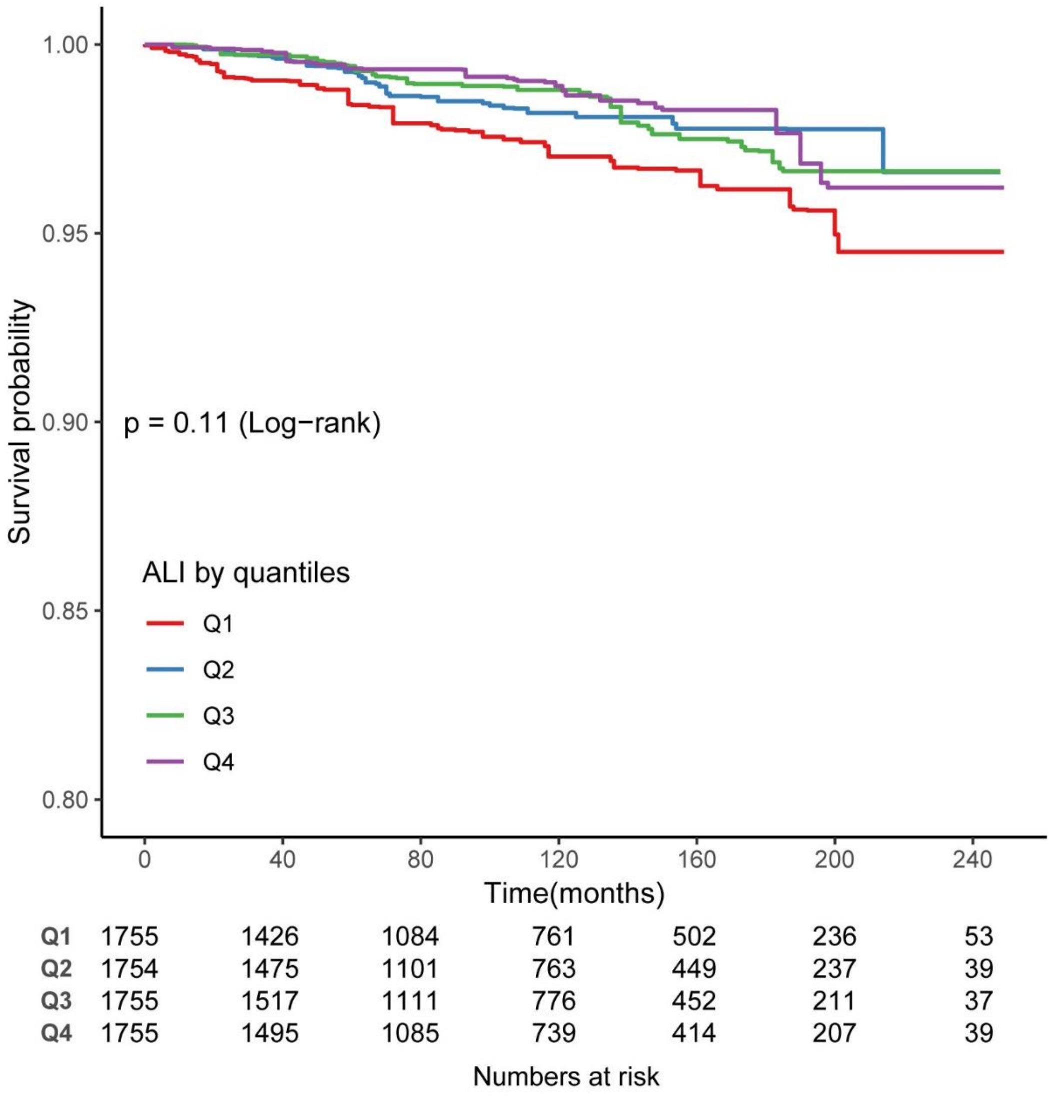

Supplementary Figure S1. Kaplan-Meier survival curve for cancer mortality. In the Kaplan-Meier curves, the population is stratified into four groups (Q1, Q2, Q3, Q4) based on the quartiles of ALI, and statistical analysis is conducted using the log-rank test

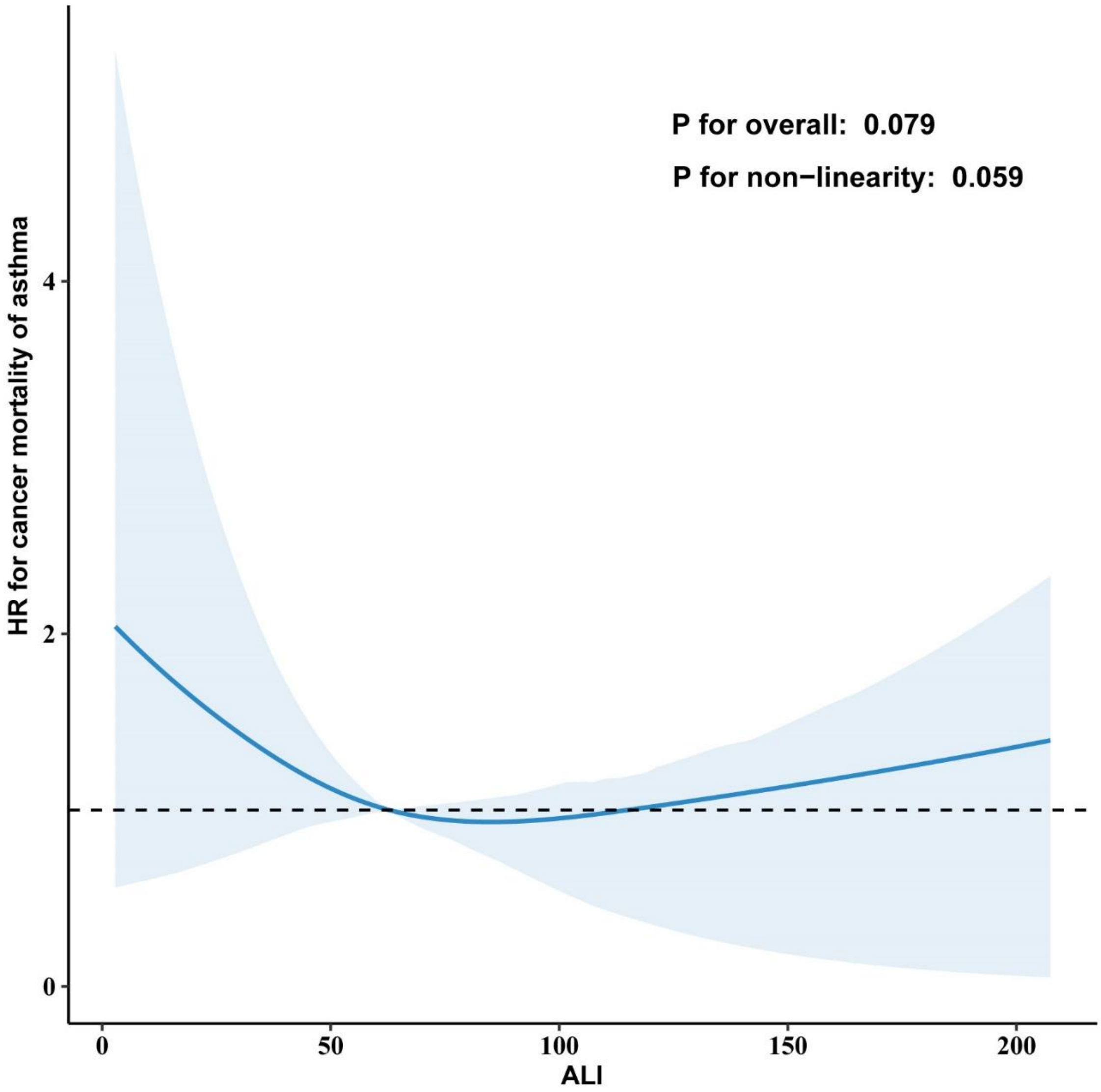

Supplementary Figure S1. Restricted cubic spline fitting for the association between ALI with cancer mortality. Only 99% of the data is shown.

Supplementary Table S4. Association of ALI with All-Cause, CVD, and Cancer Mortality in Asthma Patients from the NHANES Cohort, Excluding ASCVD Population (**n=6145**)

ALI	All -cause mortality						
	**Events**	**Crude Model**		**Model 1**		**Model 2**	
		**HR, 95%CI**	***p*-Value**	**HR, 95%CI**	***p*-Value**	**HR, 95%CI**	***p*-Value**
**Per10Uincrement**	586(9.5)	0.92(0.87-0.96)	<0.001	0.95(0.90-0.99)	0.018	0.95(0.91-0.99)	0.022
Quantile 1	232(15.5)	1(Ref)		1(Ref)		1(Ref)	
Quantile 2	130(8.4)	0.47(0.38~0.60)	<0.001	0.59(0.45~0.76)	<0.001	0.59(0.45~0.76)	<0.001
Quantile 3	130(8.4)	0.54(0.41~0.70)	<0.001	0.66(0.50~0.87)	0.003	0.68(0.51~0.91)	0.011
Quantile 4	94(6.0)	0.35(0.26~0.47)	<0.001	0.44(0.32~0.60)	<0.001	0.44(0.32~0.61)	<0.001
***P* for trend**			<0.001		<0.001		<0.001
	**CVD mortality**						
		**Crude Model**		**Model 1**		**Model 2**	
		**HR, 95%CI**	***p*-Value**	**HR, 95%CI**	***p*-Value**	**HR, 95%CI**	***p*-Value**
Per10Uincrement	125(2.0)	0.90(0.82~0.98)	0.020	0.92(0.86~1.00)	0.037	0.92(0.86~0.99)	0.032
Quantile 1	47(3.1)	1(Ref)		1(Ref)		1(Ref)	
Quantile 2	29(1.9)	0.57(0.33~1.01)	0.054	0.74(0.42~1.32)	0.314	0.76(0.41~1.42)	0.389
Quantile 3	28(1.8)	0.65(0.34~1.24)	0.187	0.84(0.44~1.57)	0.577	0.85(0.45~1.62)	0.628
Quantile 4	21(1.3)	0.39(0.21~0.75)	0.005	0.46(0.23~0.92)	0.028	0.46(0.23~0.89)	0.022
***P* for trend**			0.015		0.068		0.058
	**Cancer mortality**						
		**Crude Model**		**Model 1**		**Model 2**	
		**HR, 95%CI**	***p*-Value**	**HR, 95%CI**	***p*-Value**	**HR, 95%CI**	***p*-Value**
Per10Uincrement	125(2.0)	0.96(0.88~1.05)	0.423	0.99(0.93~1.05)	0.752	0.99(0.95~1.04)	0.732
Quantile 1	36(2.4)	1(Ref)		1(Ref)		1(Ref)	
Quantile 2	32(2.1)	0.68(0.39~1.16)	0.155	0.85(0.48~1.51)	0.574	0.87(0.49~1.54)	0.639
Quantile 3	35(2.3)	0.71(0.40~1.28)	0.256	0.92(0.50~1.69)	0.780	0.98(0.54~1.79)	0.957
Quantile 4	22(1.4)	0.46(0.23~0.94)	0.032	0.60(0.29~1.23)	0.163	0.65(0.31~1.34)	0.240
***P* for trend**			0.043		0.248		0.371

Notes: Crude Model: no covariates were adjusted;
Model 1: Adjusted for gender, age, race, education level, marital status, PIR;
Model 2 was adjusted for model 1+ smoking status, alcohol use, hypertension, diabetes, cancer, eosinophils, chronic bronchitis.
Abbreviations: ALI advanced lung cancer inflammation index; CVD: Cardiovascular Disease; PIR: Ratio of family income to poverty; BMI body mass index; Ref：Reference;

Supplementary Table S5. Association of ALI with All-Cause, CVD, and Cancer Mortality in Asthma Patients from the NHANES Cohort, Excluding cancer population (**n=6332**)

**ALI**	**All -cause mortality**						
	**Events**	**Crude Model**		**Model 1**		**Model 2**	
		**HR, 95%CI**	***p*-Value**	**HR, 95%CI**	***p*-Value**	**HR, 95%CI**	***p*-Value**
**Per10Uincrement**	684(10.8)	0.92(0.88-0.95)	<0.001	0.95(0.92-0.98)	0.004	0.95(0.92-0.98)	0.002
Quantile 1	265(17.3)	1(Ref)		1(Ref)		1(Ref)	
Quantile 2	160(10.0)	0.55(0.44~0.68)	<0.001	0.68(0.54~0.85)	<0.001	0.68(0.56~0.84)	<0.001
Quantile 3	147(9.2)	0.54(0.42~0.70)	<0.001	0.68(0.51~0.89)	0.006	0.69(0.52~0.92)	0.012
Quantile 4	112(7.0)	0.40(0.31~0.53)	<0.001	0.54(0.41~0.71)	<0.001	0.51(0.39~0.68)	<0.001
***P* for trend**			<0.001		<0.001		<0.001
	**CVD mortality**						
		**Crude Model**		**Model 1**		**Model 2**	
		**HR, 95%CI**	***p*-Value**	**HR, 95%CI**	***p*-Value**	**HR, 95%CI**	***p*-Value**
Per10Uincrement	177(2.8)	0.89(0.83~0.96)	0.002	0.93(0.87~0.99)	0.025	0.92(0.86~0.98)	0.007
Quantile 1	65(4.3)	1(Ref)		1(Ref)		1(Ref)	
Quantile 2	46(2.9)	0.72(0.47~1.11)	0.134	0.95(0.61~1.48)	0.827	0.92(0.60~1.42)	0.711
Quantile 3	40(2.5)	0.74(0.43~1.27)	0.274	0.96(0.55~1.69)	0.892	0.91(0.52~1.59)	0.742
Quantile 4	26(1.6)	0.35(0.20~0.62)	<0.001	0.46(0.25~0.84)	0.012	0.40(0.23~0.71)	0.002
***P* for trend**			0.002		0.063		0.013

Notes: Crude Model: no covariates were adjusted;
Model 1: Adjusted for gender, age, race, education level, marital status, PIR;
Model 2 was adjusted for model 1+ smoking status, alcohol use, hypertension, diabetes, ASCVD, eosinophils, chronic bronchitis.
Abbreviations: ALI advanced lung cancer inflammation index; CVD: Cardiovascular Disease; PIR: Ratio of family income to poverty; BMI body mass index; Ref：Reference;

Supplementary Table S6. Association of ALI with All-Cause, CVD, and Cancer Mortality in Asthma Patients from the NHANES Cohort, Excluding diabetes population (**n=6052**)

**ALI**	**All -cause mortality**						
	**Events**	**Crude Model**		**Model 1**		**Model 2**	
		**HR, 95%CI**	***p*-Value**	**HR, 95%CI**	***p*-Value**	**HR, 95%CI**	***p*-Value**
**Per10Uincrement**	640(10.6)	0.91(0.86~0.96)	<0.001	0.95(0.91~0.99)	0.029	0.96(0.91~1.00)	0.048
Quantile 1	271(17.7)	1(Ref)		1(Ref)		1(Ref)	
Quantile 2	141(9.2)	0.48(0.38~0.61)	<0.001	0.65(0.50~0.84)	0.001	0.66(0.52~0.84)	<0.001
Quantile 3	131(8.7)	0.51(0.39~0.66)	<0.001	0.63(0.49~0.82)	<0.001	0.66(0.51~0.86)	0.002
Quantile 4	97(6.5)	0.32(0.25~0.42)	<0.001	0.45(0.35~0.59)	<0.001	0.48(0.36~0.62)	<0.001
***P* for trend**			<0.001		<0.001		<0.001
	**CVD mortality**						
		**Crude Model**		**Model 1**		**Model 2**	
		**HR, 95%CI**	***p*-Value**	**HR, 95%CI**	***p*-Value**	**HR, 95%CI**	***p*-Value**
Per10Uincrement	154(2.5)	0.90(0.83~0.97)	0.008	0.95(0.89~1.01)	0.088	0.95(0.89~1.01)	0.097
Quantile 1	56(3.7)	1(Ref)		1(Ref)		1(Ref)	
Quantile 2	37(2.4)	0.66(0.40~1.09)	0.108	1.00(0.60~1.65)	0.985	1.00(0.61~1.63)	0.989
Quantile 3	39(2.6)	0.81(0.48~1.38)	0.445	1.09(0.66~1.80)	0.749	1.12(0.69~1.84)	0.640
Quantile 4	22(1.5)	0.33(0.19~0.59)	<0.001	0.50(0.27~0.92)	0.026	0.50(0.27~0.94)	0.031
***P* for trend**			0.006		0.173		0.198
	**Cancer mortality**						
		**Crude Model**		**Model 1**		**Model 2**	
		**HR, 95%CI**	***p*-Value**	**HR, 95%CI**	***p*-Value**	**HR, 95%CI**	***p*-Value**
Per10Uincrement	141(2.3)	1.00(0.91~1.10)	0.989	1.01(0.98~1.03)	0.521	1.01(0.99~1.03)	0.557
Quantile 1	47(3.1)	1(Ref)		1(Ref)		1(Ref)	
Quantile 2	31(2.0)	0.52(0.31~0.88)	0.014	0.69(0.39~1.22)	0.206	0.70(0.41~1.22)	0.211
Quantile 3	36(2.4)	0.64(0.37~1.11)	0.112	0.84(0.48~1.47)	0.544	0.85(0.49~1.48)	0.575
Quantile 4	27(1.8)	0.47(0.25~0.91)	0.025	0.66(0.34~1.29)	0.224	0.72(0.37~1.41)	0.336
***P* for trend**			0.038		0.296		0.403

Notes: Crude Model: no covariates were adjusted;
Model 1: Adjusted for gender, age, race, education level, marital status, PIR;
Model 2 was adjusted for model 1+ smoking status, alcohol use, hypertension, ASCVD, cancer, eosinophils, chronic bronchitis.

Abbreviations: ALI advanced lung cancer inflammation index; CVD: Cardiovascular Disease; PIR: Ratio of family income to poverty; BMI body mass index; Ref：Reference;

Supplementary Table S7. Association between ALI and Cardiovascular Mortality using Fine & Gray Competing Risks Models

**ALI**	**CVD mortality**				
	**Number of deaths**	**Unadjusted model**		**Multivariable adjusted model**	
		**SHR, 95%CI**	***p*-Value**	**SHR, 95%CI**	***p*-Value**
Per10Uincrement	220(3.1)	0.90(0.85~0.95)	<0.001	0.95(0.92~1.00)	0.029
Quantile 1	85(4.8)	1(Ref)		1(Ref)	
Quantile 2	54(3.1)	0.66(0.47~0.94)	0.019	0.98(0.69~1.40)	0.932
Quantile 3	49(2.8)	0.60(0.42~0.86)	0.005	0.93(0.65~1.33)	0.686
Quantile 4	32(1.8)	0.41(0.27~0.61)	<0.001	0.67(0.43~1.03)	0.065
***P* for trend**			<0.001		0.086

Notes: Unadjusted model: no covariates were adjusted; **Multivariable adjusted model**: Adjusted for gender, age, race, education level, marital status, PIR, smoking status, alcohol use, ASCVD, hypertension, diabetes, cancer, eosinophils, chronic bronchitis.

Supplementary Table S8. Multivariable Analysis of the Association Between ALI and All-Cause, CVD, and Cancer Mortality After Exclusion of Extreme Values (**n=6958**)

ALI	All -cause mortality						
	**Events**	**Crude Model**		**Model 1**		**Model 2**	
		**HR, 95%CI**	***p*-Value**	**HR, 95%CI**	***p*-Value**	**HR, 95%CI**	***p*-Value**
**Per10Uincrement**	877(12.6)	0.89(0.86~0.92)	<0.001	0.93(0.90~0.96)	<0.001	0.93(0.90~0.96)	<0.001
Quantile 1	359(20.5)	1(Ref)		1(Ref)		1(Ref)	
Quantile 2	200(11.4)	0.52(0.42~0.63)	<0.001	0.66(0.54~0.82)	<0.001	0.67(0.55~0.82)	<0.001
Quantile 3	183(10.4)	0.51(0.41~0.64)	<0.001	0.65(0.51~0.82)	<0.001	0.65(0.50~0.84)	0.001
Quantile 4	135(8.0)	0.38(0.30~0.47)	<0.001	0.52(0.40~0.67)	<0.001	0.51(0.40~0.66)	<0.001
***P* for trend**			<0.001		<0.001		<0.001
	**CVD mortality**						
		**Crude Model**		**Model 1**		**Model 2**	
		**HR, 95%CI**	***p*-Value**	**HR, 95%CI**	***p*-Value**	**HR, 95%CI**	***p*-Value**
Per10Uincrement	220(3.2)	0.89(0.83~0.96)	0.002	0.94(0.88~1.00)	0.064	0.94(0.88~1.00)	0.043
Quantile 1	85(4.8)	1(Ref)		1(Ref)		1(Ref)	
Quantile 2	54(3.1)	0.63(0.43~0.93)	0.019	0.87(0.58~1.29)	0.475	0.86(0.58~1.28)	0.452
Quantile 3	49(2.8)	0.68(0.42~1.09)	0.110	0.93(0.57~1.51)	0.769	0.90(0.55~1.48)	0.679
Quantile 4	32(1.9)	0.37(0.22~0.60)	<0.001	0.54(0.32~0.90)	0.018	0.50(0.29~0.85)	0.011
***P* for trend**			<0.001		0.089		0.045
	**Cancer mortality**						
		**Crude Model**		**Model 1**		**Model 2**	
		**HR, 95%CI**	***p*-Value**	**HR, 95%CI**	***p*-Value**	**HR, 95%CI**	***p*-Value**
Per10Uincrement	176(2.5)	0.92(0.85~1.00)	0.037	0.96(0.90~1.03)	0.261	0.97(0.91~1.03)	0.364
Quantile 1	60(3.4)	1(Ref)		1(Ref)		1(Ref)	
Quantile 2	42(2.4)	0.57(0.36~0.92)	0.020	0.73(0.45~1.19)	0.209	0.76(0.47~1.23)	0.270
Quantile 3	43(2.5)	0.57(0.35~0.94)	0.027	0.75(0.45~1.24)	0.260	0.75(0.44~1.28)	0.294
Quantile 4	31(1.8)	0.49(0.27~0.91)	0.023	0.68(0.36~1.27)	0.227	0.75(0.40~1.40)	0.369
***P* for trend**			0.018		0.198		0.298

Notes: Crude Model: no covariates were adjusted;
Model 1: Adjusted for gender, age, race, education level, marital status, PIR;
Model 2 was adjusted for model 1+ smoking status, alcohol use, hypertension, diabetes, cancer, eosinophils, ASCVD, chronic bronchitis.
Abbreviations: ALI advanced lung cancer inflammation index; CVD: Cardiovascular Disease; PIR: Ratio of family income to poverty; BMI body mass index; Ref：Reference;

Supplementary Table S9. Multivariable Analysis of the Association Between ALI and All-Cause, CVD, and Cancer Mortality After Exclusion of Missing Values (**n=4829**)

**ALI**	**All -cause mortality**						
	**Events**	**Crude Model**		**Model 1**		**Model 2**	
		**HR, 95%CI**	***p*-Value**	**HR, 95%CI**	***p*-Value**	**HR, 95%CI**	***p*-Value**
**Per10Uincrement**	582(12.1)	0.89(0.83~0.94)	<0.001	0.94(0.89~0.99)	0.030	0.93(0.88~0.98)	0.013
Quantile 1	244(20.2)	1(Ref)		1(Ref)		1(Ref)	
Quantile 2	129(10.7)	0.48(0.38~0.62)	<0.001	0.69(0.52~0.91)	0.009	0.64(0.49~0.83)	<0.001
Quantile 3	121(10.0)	0.45(0.33~0.61)	<0.001	0.63(0.46~0.87)	0.005	0.58(0.42~0.80)	<0.001
Quantile 4	88(7.3)	0.32(0.24~0.42)	<0.001	0.47(0.34~0.65)	<0.001	0.42(0.30~0.57)	<0.001
***P* for trend**			<0.001		<0.001		<0.001
	**CVD mortality**						
		**Crude Model**		**Model 1**		**Model 2**	
		**HR, 95%CI**	***p*-Value**	**HR, 95%CI**	***p*-Value**	**HR, 95%CI**	***p*-Value**
Per10Uincrement	151(3.1)	0.85(0.77~0.95)	0.003	0.92(0.84~1.00)	0.061	0.90(0.82~0.99)	0.032
Quantile 1	65(5.4)	1(Ref)		1(Ref)		1(Ref)	
Quantile 2	37(3.1)	0.52(0.32~0.82)	0.005	0.81(0.49~1.34)	0.408	0.72(0.46~1.14)	0.166
Quantile 3	29(2.4)	0.44(0.24~0.82)	0.009	0.69(0.37~1.30)	0.253	0.62(0.33~1.16)	0.136
Quantile 4	20(1.7)	0.27(0.15~0.51)	<0.001	0.45(0.24~0.87)	0.018	0.38(0.19~0.74)	0.005
***P* for trend**			<0.001		0.033		0.008
	**Cancer mortality**						
		**Crude Model**		**Model 1**		**Model 2**	
		**HR, 95%CI**	***p*-Value**	**HR, 95%CI**	***p*-Value**	**HR, 95%CI**	***p*-Value**
Per10Uincrement	125(2.6)	0.98(0.85~1.13)	0.800	1.01(0.94~1.08)	0.788	1.01(0.95~1.08)	0.762
Quantile 1	43(3.6)	1(Ref)		1(Ref)		1(Ref)	
Quantile 2	29(2.4)	0.47(0.27~0.85)	0.011	0.66(0.35~1.25)	0.204	0.61(0.33~1.16)	0.131
Quantile 3	31(2.6)	0.52(0.30~0.92)	0.024	0.76(0.42~1.37)	0.359	0.69(0.37~1.29)	0.250
Quantile 4	22(1.8)	0.33(0.16~0.67)	0.002	0.48(0.23~1.02)	0.056	0.48(0.23~1.01)	0.053
***P* for trend**			0.003		0.081		0.072

Notes: Crude Model: no covariates were adjusted;
Model 1: Adjusted for gender, age, race, education level, marital status, PIR;
Model 2 was adjusted for model 1+ smoking status, alcohol use, hypertension, diabetes, cancer, eosinophils, ASCVD, chronic bronchitis.
Abbreviations: ALI advanced lung cancer inflammation index; CVD: Cardiovascular Disease; PIR: Ratio of family income to poverty; BMI body mass index; Ref：Reference;
